# Determinants of the implementation of eHealth-based long-term follow-up care for young cancer survivors: a qualitative study

**DOI:** 10.1186/s12885-024-12910-6

**Published:** 2024-09-18

**Authors:** Tjorven Stamer, Pia Traulsen, Johannes Rieken, Teresa Schmahl, Ingo Menrath, Jost Steinhäuser

**Affiliations:** 1 Institute of Family Medicine, UKSH Luebeck, Ratzeburger Allee 160, 23562 Luebeck, Germany; 2 Clinic of Pediatric and Adolescent Medicine, UKSH Luebeck, Ratzeburger Allee 160, 23562 Luebeck, Germany

**Keywords:** eHealth, Telemedicine, Cancer, Oncology, Children, General practice, Qualitative research, Implementation science

## Abstract

**Background:**

eHealth may help closing gaps in the long-term follow-up care of former young age cancer patients. While its introduction to medical aftercare appears promising, it also faces obstacles in the course of its implementation. This study explored what prospective eHealth applications have to achieve and what facilitating and hindering factors are associated with the implementation of them.

**Methods:**

A qualitative, explorative-descriptive design involving semi-structured interviews was used in this study. General practitioners (GPs) from urban and rural areas as well as former cancer patients were recruited and interviewed. The interview guide focused on expectations of telemedical care services for the patient group of children and adolescents as well as potential facilitating and hindering factors of the implementation of telemedical care services for former cancer patients. Interviews were recorded, transcribed and analyzed on the basis of qualitative content analysis as described by Kuckartz.

**Results:**

Empiric saturation was reached after 25 interviews, respectively. The age of the physicians surveyed at the time of the interviews ranged from 27 to 71 years, with an average of 42 years. The former patients ranged in age from 21 to 43 at the time of participation, with an average age of 34. The age at diagnosis ranged from 3 to 31 years. eHealth services were considered an effective way to maintain continuity of care and improve the health literacy of cancer survivors. Cooperation with health insurance companies and gamification-elements were regarded as beneficial for the introduction of eHealth structures. Poor interface compatibility, insufficient network coverage and lack of digital literacy were valued as potential barriers.

**Conclusions:**

If properly introduced, eHealth shows the potential to provide stakeholders with tools that increase their self-efficacy and ability to act. As the technology continues to advance, our data provides application-oriented factors for tailored implementation strategies to bring eHealth into the field.

**Supplementary Information:**

The online version contains supplementary material available at 10.1186/s12885-024-12910-6.

## Background

Worldwide, approximately 280.000 children and adolescents between the ages of 0 and 19 are diagnosed with cancer every year [1, 2]. Despite increasing survival rates of around 80%, the majority of young people with cancer suffer from late effects and complications of the disease and its treatment [3]. These include e.g. a new oncologic disease or myocardial insufficiency [3, 4]. In addition, the issue of infertility is of great importance, as oncological treatment is associated with an increased risk of infertility [5]. Prevalence of late complications rises with increasing temporal distance to the primary oncological disease and therapy, not reaching a plateau effect even decades upon treatment [5].

This resulted in medical aftercare recommendations aiming at the early detection and treatment of these diseases on the basis of lifelong, risk adapted preventive and long-term follow up care programs. For patients undergoing regular medical aftercare, late complications get detected earlier and hospital stays are reduced [6, 7]. These patients possess a better understanding as well as knowledge about their disease and show a higher health-related self-efficacy [8]. In conclusion, a risk-adapted long-term follow-up care can reduce mortality and morbidity in cancer survivors [9, 10]. Likewise, improvements regarding health-related quality of life have been associated with early and long-term follow-up care of former cancer patients [11].

### Long-term follow-up care

Long-term follow-up care for former cancer patients refers to the organized process of monitoring as well as navigating the long-term post treatment stage, starting upon the end of active treatment [12, 13].

Upon active treatment, recovered children usually are in aftercare programs in children’s oncology departments [14, 15]. Around the age of 18, they commonly undergo a transition to long-term follow-up care, which is usually provided by oncologists in collaboration with General practitioners (GPs) [15]. Individual situations must be considered regarding the exact age of transfer to a more age-appropriate provider [14–16].

However, the patient group beyond the age of 18 often perceives itself as cured and “healthy”, leading, among other reasons, to a low participation rate in aftercare [16–18]. Currently, a total of 1000 patients per year are treated in German long-term follow-up care centers, which represents about 3% of all cancer survivors at a young age, i.e. in childhood or adolescence [18]. Furthermore, there is a lack of needs-based patient information and participatory care design as well as evaluated, structured training in the sense of *Patient Empowerment*, especially in the transition phase from pediatric to adult medicine [18, 19].

### Clinical practice guidelines

Different guidelines directing clinical practice for the long-term follow-up care of cancer survivors are existing, such as recommendations from the International Late Effects of Childhood Cancer Guideline Harmonization Group (IGHG) or guidelines from the Association of the Scientific Medical Societies in Germany (AWMF) [20, 21].

The IGHG offers a list of relevant publications as well as numerous specific recommendations for practice [20].

In Germany, a guideline was proposed by the AWMF, namely the *S1-guideline for the long-term follow-up care of children*,* adolescents and young adults with cancer – Avoiding*,* recognizing and treating late effects* [21]. This guideline was established for providing treatment guidance for young cancer survivors with oncological and hematological diseases.

### eHealth

The term *eHealth*, also referred to as *digital health*, is used for the application of electronic and internet-based systems to support healthcare delivery [22, 23]. One component of it is *telemedicine*, also referred to as *telehealth*, which may be defined as “the use of information and communication technologies to deliver healthcare services at a distance” [24]. Correspondingly, it is regarded as an effective and efficient tool to provide comprehensive access to appropriate care as needed [25]. In the recent past, the use of eHealth-based and telemedical care services has seen a major increase, to some point boosted by the global SARS-CoV-2 pandemic as well as the general advance of digitalization [26]. However, the long-term adoption rates remained low [27]. Still, in the context of long-term follow-up care for young cancer survivors, it may serve as a useful supplement to address certain gaps in medical services, such as the inability to reach underserved rural and remote cancer survivor populations or poor asynchronous communication among multiple clinicians and their patients [28, 29]. This *teleoncology* is employed to improve cancer patients’ access to care by lowering the requirement to travel to distant oncology centers, resulting in saving time, effort and costs [30, 31]. The use of eHealth and telemedicine also faces barriers in the course of its implementation. Examples for this are financial constraints to establish the required technological infrastructure [32] as well as the protection of patients’ data privacy [33, 34].

### General practitioners’ involvement in cancer care

In this study, we’re focusing on the role of GPs and former cancer patients in the medical aftercare of cancer survivors, since GPs are involved in the care of 90% of all chronic medical conditions [35]. This is comprehensible, since 90% of patients have a GP and have contact at least once a year [36]. 80% of all reasons for encounter can usually be solved by a GPs [36]. As such, an early integration of the GPs in the comprehensive cancer treatment may enhance patient’s trust and psychosocial well-being [37]. Furthermore, the GP’s office in most cases serves as the first point of contact as well as guidance for young cancer patients, as the GP possesses a vital role in providing diagnosis, direct health care and further orientation [38–40].

### Implementation science

We pursue an implementation science approach. Implementation science is the scientific study of methods to promote the systematic adoption of research findings and other evidence-based practices into routine practice, with the aim of improving the quality and effectiveness of healthcare services [41]. Serving as an orientation for this transition process, its principles focus on the identification of aspects that directly influence the introduction of new applications and methods, i.e. the facilitating and hindering factors [42]. Various instruments are available in this context, e.g. the *Behavior Change Wheel (BCW)*, a tool to tailor implementation strategies to targeted scenarios [43]. Working as a framework for the development of behavior change interventions, the BCW guides decision-making and choice of appropriate intervention for the desired implementation process.

### Research questions

This study explores the determinants of successfully implementing eHealth in the long-term follow-up care of young cancer survivors.

## Methods

This study is part of the *LaNCa* (*Long-term follow-up care after cancer in childhood*,* adolescence and young adulthood in Schleswig-Holstein - New care services on the topic of “Cancer Survivorship”*) research project (registration number DRKS00027264), funded by the Ministry of Social Affairs, Health, Youth, Family and Senior Citizens of the state of Schleswig-Holstein. *LaNCa* aims to establish a better, cross-sectoral medical aftercare for young cancer survivors and thus to translate scientific findings into practice.

The Consolidated Criteria for Reporting Qualitative Research (COREQ) guidelines have been followed (supplementary file 1) [44].

### Design and interview guide

A qualitative exploratory-descriptive study design involving semi-structured interviews was chosen to address our research questions. Two separate interview guides were created for the two participating groups of general practitioners and former patients. The development of the two interview guides was essentially based on the research question of the determinants of successful implementation of eHealth in long-term follow-up care after cancer at a young age. As part of the preparation process, the research team’s existing expertise on the topic was used [45–47]. The semi-structured interview guides comprised eight and six questions, respectively, inquiring about experiences, motivational aspects of the target group to participate in research projects, expectations of telemedical care services for this domain, facilitating and hindering factors of the implementation of telemedical care services for cancer patients as well as facilitating and hindering factors in relation to guideline-compliant care (supplementary file 2 and 3).

### Study population and eligibility criteria

We conducted interviews with 25 GPs working in either urban or rural areas. Participants were eligible for the interviews if they were either working as GP in a general practice or were in postgraduate training to become a GP. Furthermore, 25 interviews with former patients were conducted. Patients were eligible if they had a history of oncologic disease in childhood, adolescence or young adulthood and had completed their corresponding therapy. The commonly used age limit of 35 years was used to define the upper limit of young adulthood [48]. Patients had to be at least 18 years of age at interview participation.

### Sampling and recruiting

The sampling of the GPs was conducted with support of the teaching practice network of the Institute of Family Medicine at the University Medical Centre Schleswig-Holstein, Luebeck. GPs in the practice network were made aware of the study at a joint research event and invited to participate in an interview if they were interested. In addition, convenience sampling was used with the help of general practitioners affiliated with the institute, who were contacted and invited to participate after giving their consent. Sampling was carried out until no additional data was being found whereby properties of the overarching categories could have been developed, i.e. empiric data saturation was reached. In the context of qualitative research, data saturation refers to the extent to which new data replicate what was contained in previously collected data [49]. As such, it is the most common means of assessing the appropriateness of sample size in qualitative research. The concept of data saturation is congruent with the Kuckartz approach to data analysis, which we have chosen to use [50].

Former patients were identified through the Schleswig-Holstein State Cancer Registry. Eligible participants were contacted directly by the first author of this paper (TSt - Psychologist) and, upon providing written informed consent, invited to an interview. As with GPs, sampling was continued until empirical data saturation was reached.

### Data collection

The interviews were conducted between April 2022 and December 2023 for the target group of the GPs and between November 2023 and February 2024 for the group of cancer survivors. GPs and patients were individually interviewed by telephone or face-to-face by TSt using the semi-structured interview guides.

### Data analysis

**Fig. 1:**
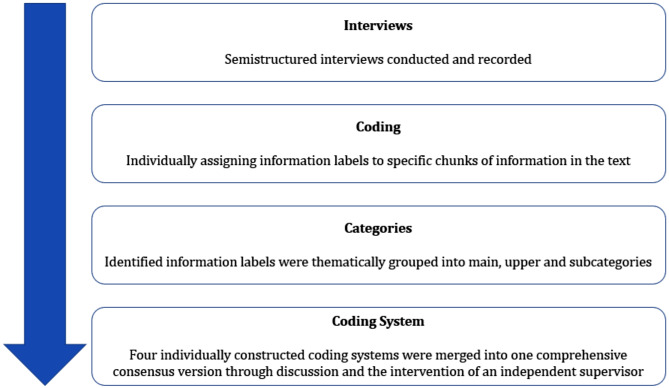
Graphic overview of the course of the qualitative approach, based on Kuckartz [50]

Interview recordings were transcribed and pseudonymized by research assistants of the Institute of Family Medicine at the University Medical Centre Schleswig-Holstein, Luebeck according to the rules of the Institute. Interviews were then coded and analyzed along the guidelines of the structuring qualitative content analysis by Kuckartz [50]. Deductively formed categories derived from the interview guide were supplemented by inductively formed categories based on the identified coded data from the transcribed interviews. Coding and content analysis were conducted by the stated authors of this study, who are researchers of the Institute of Family Medicine (TSt - psychologist, PT – health services researcher, JR – general practitioner and TS – speech and language therapist). TSt, PT, JR and TS independently coded the data of the interviews. This was done by assigning descriptive labels to specific passages in the text that were relevant to the aforementioned research questions. Identified data labels were then thematically grouped into main, secondary and subcategories, forming a comprehensive coding system. Subcategories at the system, patient and physician level were created, guided by the corresponding levels to be included in the sense of a fruitful and efficient implementation process [51]. This process was inductive as well as deductive in nature, since good implementation practice guided the levels to be included, while information taken directly from the transcribed interviews was supporting it [52]. Upon creating four individually constructed coding systems, the researchers compared their work to arrive at a consensus version. Any disagreements that arose were resolved through discussion with and the intervention of a fifth researcher (JS – general practitioner, experienced in qualitative research) who acted as supervisor (Fig. 1).

This qualitative data analysis procedure was followed for both the interviews with the general practitioners and the interviews with the former patients, resulting in two different coding systems. We conducted investigator triangulation, in which different multidisciplinary team members independently analyzed the data and then compared interpretations to ensure consistency. This multifaceted approach allowed us to gain a comprehensive understanding of the data.

## Results

After conducting 25 interviews with GPs, empiric data saturation was reached. 9 participants identified as female, 16 as male. 10 participants were in postgraduate medical training. The average age was 42 years (min: 27, max: 71). Median for the interview time in minutes was 24.

25 interviews were conducted with former patients until empiric data saturation was reached. 13 identified as female, 12 as male. The average age was 34 years (min: 21, max: 43). The mean age at diagnosis was 18 years (min: 3, max: 31). Median for the interview time in minutes was 18.

### Facilitating factors for the use of telemedical care services – general practitioners

Facilitating factors brought up by the GPs encompassed the maintenance of a continuity of care due to permanent access to medical help and tailored access to care, for instance synchronous as well as asynchronous contact options.

*“But the aim is somehow to improve the therapy and so that the resumption or the intervals at which contact is made again and possibly the optimum time for checks*,* be it laboratory checks or X-ray checks*,* so that this is not missed.” (Physician #9)*.

GPs considered telemedical care services to support the health literacy of cancer survivors, providing them with a sense of self-efficacy and further supporting them within their own treatment.

*“Patients are responsible for themselves*,* but you want them to be adherent themselves in the sense of shared decision making in the form of consent*,* you always want to strengthen patients’ health literacy*,* also in the context of aftercare.” (Physician #25)*.

Furthermore, technical aspects, such as technical support, a compatibility with existing practice management systems as well as a cross-devise and location-independent use were named.

*“At best*,* it has to be compatible with the software I’m working with anyway.” (Physician #15)*.

Further facilitating factors are shown in Table 1.

**Table 1:** Thematically structured categorical system for the physician’s sample – facilitating factors

Category	Subcategory	Quote
System level	• Compatibility with existing practice management system	• *“At best, it has to be compatible with the software I’m working with anyway.” (Physician #15)*
	• Technical support	• *“There must be someone who makes these appointments and someone who is familiar with them if there are any problems that can be addressed.” (Physician #2)*
	• Smart functions	• *“If the patient has any things in the file*,* height*,* weight or whatever I need*,* blood pressure*,* then it has to be automatically transferred from the file into the tool.” (Physician #25)*
	• Cross-device use	• *“Not having a corresponding device that goes with it*,* but that you can use it on many possible devices.” (Physician #17)*
	• Trainings	• *“There must be training on how to do this.” (Physician #3)*
	• Location-independent use	• *“And it has to be easy to install everywhere*,* not only on my practice software*,* also at home*,* if necessary.” (Physician #15)*
	• Free of charge	• *“Yes*,* of course it has to be accessible to patients*,* free of charge*,* for me*,* too*,* in the best-case scenario.” (Physician #14)*
	• Information privacy	• *“I think for me the biggest thing is always whether you are afraid of data misuse or not.” (Physician #13)*
	• High Usability	• *“It should be user-friendly for both sides*,* i. e. for the department and above all for the participants.” (Physician #21)*
Patient level	• Saving time and effort	• *“People don’t have to invest the time to leave the house and drive to a practice.” (Physician #22)*
	• Supporting further treatment	• *“For the patient to keep a good overview for themselves and not so doctor-centered but more patient-centered.” (Physician #14)*
	• Supporting health literacy	• *“Patients are responsible for themselves*,* but you want them to be adherent themselves in the sense of shared decision making in the form of consent*,* you always want to strengthen patients’ health literacy*,* also in the context of aftercare.” (Physician #25)*
	• 24/7 access to care	• *“It’s like “I have a quick question” and someone gives you feedback or you have a psychological stress situation that arises and then have another short conversation.” (Physician #21)*
	• Familiar contact	• *“Behind the system there is also a person who they may know or maybe he has made a personalized video and it is always the same person.” (Physician #25)*
	• Continuity of care	• *“But the aim is somehow to improve the therapy and so that the resumption or the intervals at which contact is made again and possibly the optimum time for checks*,* be it laboratory checks or X-ray checks*,* so that this is not missed.” (Physician #9)*
	• Perceived added value	• *“When you can actually establish a link - we want to measure something now - that is the benefit for you.” (Physician #19)*
	• Saving money	• *“So that it simply saves trips and travel costs*,* I think that would certainly be a positive factor.” (Physician #7)*
	• Digital literacy	• *“So*,* I believe that we won’t have the problem that people don’t know how to use a digital platform that is somehow structured like a usual platform these days.” (Physician #23)*
	• Low-threshold access	• *“So clearly*,* a very low-threshold approach.” (Physician #21)*
	• Higher flexibility	• *“I don’t know*,* the participants live in the countryside and it’s great if there is a talking video device*,* also a practice team that can use the electronic stethoscope*,* the electronic otoscope*,* etc. for the patients.” (Physician #20)”*
	• Gamification elements	• *“Gamification: bees*,* stars*,* bling bling*,* well done*,* great*,* reminders. Then anything that’s educational*,* even if it’s packed into a quiz.” (Physician #25)*
	• Supported by health insurance	• *“Support in the sense of how health insurance companies offer it without imposing it*,* which is a fine line.” (Physician #19)*
Physician level	• Early inclusion of the general practitioner in decision-making	• *“If you were involved in the decision-making process for your patient.” (Physician #2)*
	• Visual impression • Reduced transmission of infection within the medical practice	• *“I would also like it to be via video conference or something like that*,* so that you don’t work too much on the electronic level and perhaps don’t just feel like processed data*,* but that the personal aspect of medicine is still there.” (Physician #17)*
	• Low-threshold access to specialist knowledge	• *“Low-threshold contact access between specialists and general practitioners is also a factor for digitalization.” (Physician #22)*
	• Optimized monitoring	• *“It can then be checked to see what has to be done when and how*,* that it is easier for the doctor to see it in one app so that the patient doesn’t have to search around for so long and can simply record everything in this one app. " (Physician #14)*
	• Work facilitation	• *“For me as a doctor*,* it’s similar when I do telemedicine*,* I don’t have to go to work*,* maybe I don’t even have to book a room for the day I don’t have to pay room rent. I know some practices with severe staff shortages that are switching to telemedicine. I am more flexible myself” (Physician #20)*
	• Adequate remuneration	• *“Honestly*,* compensation*,* that sounds totally stupid*,* but I can see how*,* there are so many budget cuts at the moment*,* you concentrate on what brings money as a practice*,* you can find it good or bad*,* but ultimately all bosses do it and if something makes work but doesn’t bring money*,* then you don’t do it*,* that’s quite simple.” (Physician #16)*

### Hindering factors for the use of telemedical care services – general practitioners

GPs proposed several usability-related aspects such as poor usability of the application itself, a poor accessibility, e.g. for blind people and a deficient implementation in existing practice management systems. Also, for asynchronous communication services such as chats, for instance, a feeling of talking to a machine would appear to be an obstacle.

*“If it really just gives you the feeling that you’re talking to a program*,* that you might have difficulties building up the same trust in this program and telemedical care that you might have with a long-time practitioner that you know well.” (Physician #17)*.

Participants brought up an unwanted reminder of the serious oncological disease as hindering factor for the use by cancer survivors. Language barriers and a lack of sensory perceptions, such as haptics and visual cues, will do a disservice in the context of the use of telemedical care service applications as well. These circumstances, especially if amplified by a poor digital literacy, may constitute a huge barrier for the use of eHealth and telemedicine and may lead to a perceived loss of control for the physician.

*“It’s like that*,* maybe*,* loss of control is such a generic term that if you have the feeling at that moment that what you’re actually doing*,* you don’t know what you’re doing it for and why you’re doing it.” (Physician #11)*.

Furthermore, the general practitioners named the transition phase as a particular challenge in the care of this patient group.

*“Well*,* I always find that when complex things occur in childhood*,* you get the feeling when they turn eighteen that there is no longer an expert to take responsibility.” (Physician #19)*.

An overview of hindering factors cited by the GPs can be taken from Table 2.

**Table 2:** Thematically structured categorical system for the physician’s sample – hindering factors

Category	Subcategory	Quote
System level	• Insufficient network coverage	• *“Maybe you’re dependent on a good internet connection.” (Physician #16)*
	• Poor implementation in existing practice management system	• *“And I don’t think it’s actually that technically difficult*,* but I can imagine the interface problem.” (Physician #25)*
	• Technical problems	• *“Well*,* I think the thing that often doesn’t work so well in telemedicine these days is the technical conditions.” (Physician #22)*
	• Information privacy	• *“Then*,* of course*,* the internet connection was a problem - every now and then.” (Physician #24)*
	• Unclear funding	• *“Yes*,* that’s exactly the case with things like this*,* which sometimes come with really high financial pressure.” (Physician #9)*
	• Poor usability	• *“I think it’s easier to keep it short and concise then and I think it works better then.” (Physician #23)*
	• Feeling of talking to a machine	• *“If it really just gives you the feeling that you’re talking to a program*,* that you might have difficulties building up the same trust in this program and telemedical care that you might have with a long-time practitioner that you know well.” (Physician #17)*
	• Poor accessibility	• *“Yes*,* I just thought of that when you talk about barriers*,* of course someone who is blind uses their cell phone in a completely different way*,* but I think it also has to be compatible with the respective apps.” (Physician #18)*
Patient level	• Remembrance of illness	• *“On the one hand*,* of course*,* you have the fact that they want to distance themselves from their former illness and want to be normal people*,* so to speak*,* they want to live like you and me and be unrestricted. (Physician #20)*
	• Loss of control	• *“It’s like that*,* maybe*,* loss of control is such a generic term that if you have the feeling at that moment that what you’re actually doing*,* you don’t know what you’re doing it for and why you’re doing it.” (Physician #11)*
	• Language barriers	• *“Language is a problem. So*,* if we simply don’t have the same mother tongue*,* that’s always difficult with telemedicine” (Physician #24)*
	• Poor digital literacy	• *“When the first iPod came out*,* so I think it has to be an intermediate piece that*,* yes*,* it will probably be rather inhibiting for the doctors because they are not in a position to use the medium.” (Physician #14)*
Physician level	• Poor interdisciplinary communication	• *“Doctors’ letters are outrageous*,* full of abbreviations. These snooty clinicians think we know the abbreviations from every department.” (Physician #1)*
	• Poor digital literacy	• *“That’s why it has to be an intermediate step*,* it will probably be rather inhibiting for the doctors because they are not able to use it.” (Physician #15)*
	• Missing nonverbal communication	• *“Yes*,* I miss direct communication because I can also read a lot from the gestures of the person I’m talking to.” (Physician #2)*
	• Lack of haptics	• *“Yes*,* the inhibiting factors are that I can’t examine things during a phone call or video call. I’d miss the haptic aspect.” (Physician #4)*
	• Lack of olfactory perception	• *“I miss the olfactory aspect*,* which I don’t underestimate.” (Physician #5)*
	• Pharmaceutical sponsoring	• *“I think it must somehow be clear that this is not subsidized by pharmaceuticals or something else.” (Physician #9)*
	• Loss of control	• *“It’s like that*,* maybe*,* loss of control is such a generic term that if you have the feeling at that moment that what you’re actually doing*,* you don’t know what you’re doing it for and why you’re doing it.” (Physician #11)*

### Facilitating factors for the use of telemedical care services – former patients

Participants expressed that continuous access to care would be beneficial. Examples include 24/7- contact and low-threshold access to personalized treatment information. They found gamification elements to be motivating and an effective way to keep track of their own healthcare.

*“For example*,* an advent calendar*,* that you perhaps do various campaigns*,* that you keep people alive*,* that they stay active on this webpage.” (Patient #3)*.

Cooperation with health insurance companies would be beneficial for the establishment of eHealth and telemedicine services. In this context, a bonus point system with extrinsic incentives for the use of eHealth and telemedicine, e.g. combined with financial discounts, was mentioned.

*“Perhaps working together with the health insurance companies and developing a points system or getting a bonus.” (Patient #4)*.

According to the participants, eHealth and telemedicine also has the potential to support the health literacy of former patients. For example, patients could use telehealth services to learn about their own long-term care. The use of eHealth and telemedicine tailored to the individual patient would also be beneficial in terms of ensuring continuity of care. For example, contact with the usual and customary practice could be maintained.

*“Having this trust in the one GP or the one urologist or oncologist. I don’t know whether I would tell someone I’m seeing for the first time something that I might tell my GP*,* who I’ve perhaps known for 15 years. I’d meet him differently than someone I’ve now seen for the first time via telemedicine.” (Patient #25)*.

Further facilitating aspects can be found in Table 3.

**Table 3:** Thematically structured categorical system for the former patients’s sample – facilitating factors

Category	Subcategory	Quote
System level	• Stable Internet connection	• *“There needs to be good internet.” (Patient #9)*
	• Software	• *“And one or two technical things are probably also partly to do with data protection and technology. Some things are not that simple.” (Patient #15)*
	• Hardware	• *“I can bring them the CD or activate it in the portal. Both works.” (Patient #19)*
	• Accessibility	• *“Even for me*,* as I am blind. Also*,* how accessible it is.” (Patient #12)*
	• Push-Notifications	• *“For example*,* if I don’t get any push notifications that I don’t have a reminder that I’m always reminded that I have this app.” (Patient #3)*
	• Device-independent use	• *“Via cell phone*,* via iPhone*,* via tablet that it is also possible via computer. Via the three things*,* I would find that good.” (Patient #3)*
Patient level	• Saving time and effort	• *“Of course*,* I didn’t have to wait in the waiting room*,* which was very practical*,* and I didn’t have to travel either.” (Patient #3)*
	• Saving money	• *“You always have to take an overnight stay there*,* because you can’t drive back the same day.” (Patient #15)*
	• Patient-oriented language	• *“I have no idea*,* so it’s very tedious when you have to google every third word in a medical report you read because you don’t know what it means and I think that’s important.” (Patient #2)*
	• Technical support	• *“That you might have a contact person or a hotline.” (Patient #6)*
	• High Usability	• *“That not everything is always so complicated and works easily.” (Patient #2)*
	• Gamification-elements	• *“For example*,* an advent calendar*,* that you perhaps do various campaigns*,* that they stay active on this webpage.” (Patient #3)*
	• Added value	• *“That you make it very clear right from the start that it is actually an app that offers you added value.” (Patient #22)*
	• Information privacy	• *“As I said*,* data protection must of course be safe so that there is no cybercrime and security is guaranteed.” (Patient #11)*
	• Health Insurance Bonus Program	• *“Perhaps working together with the health insurance companies and developing a points system or getting a bonus.” (Patient #4)*
	• Higher flexibility	• *“It’s just right that it remains flexible - we should all always be flexible*,* so it would be nice if telemedicine also offered a bit of flexibility.” (Patient #2)*
	• Low-threshold access	• *“So*,* I’ve often had e-mail contact with [location1]*,* let’s say to exchange information or if I had a question. That was easy.” (Patient #14)*
	• Digital literacy	• *“People are creatures of habit and when habits change and you change into a new routine and get used to it*,* then this is good.” (Patient #18)*
	• 24/7-access to care	• *“I don’t know whether it’s cheeky to say working hours until midnight*,* but it would definitely ensure care.” (Patient #3)*
	• Reduced transmission of infection within the medical practice	• *“Not catching something in the waiting room that you might not want to have.” (Patient #10)*
	• Interoperability	• *“Everyone knows what video telephony is or something. So of course*,* you could do that*,* via whatever system*,* that is important. " (Patient #6)*
	• Integration of self-collected digital health data	• *“From the device or the insulin pump*,* it has been read out and the doctor can say in black and white without having to go through the diary.” (Patient #12)*
	• Patient safety	• *“I think it would definitely benefit patient safety if we could simply find a common medium*,* i. e. everything that is somehow centralized.” (Patient #9)*
	• Supporting health literacy	• *“That you also learn interesting facts about the body*,* why something is important now*,* why you have to eat a healthy diet now*,* why sugar is unhealthy*,* why you have to consume sugar*,* how you can perhaps eat better because you can control a lot with it*,* so to speak.” (Patient #3)*
	• Trainings	• *“But as I said*,* just the areas*,* so the staff should definitely be trained.” (Physician #25)*
	• Continuity of care	• *“Having this trust in the one GP or the one urologist or oncologist. I don’t know whether I would tell someone I’m seeing for the first time something that I might tell my GP*,* who I’ve perhaps known for 15 years. I’d meet him differently than someone I’ve now seen for the first time via telemedicine.” (Patient #25)*
Physician level	• Saving time	• *“That it will save them time*,* so in principle that’s probably the point behind it.” (Patient #9)*
	• General willingness to provide telemedical services	• *“I think very few doctors who work in a practice offer online consultation hours so far*,* so I think that’s really rare.” (Patient #2)*

### Hindering factors for the use of telemedical care services – former patients

Former patients mentioned technical barriers related to the hardware and software to be used. Further technical aspects comprised inadequate information privacy and insufficient broadband coverage. Participants mentioned a lack of interface compatibility, i.e. when eHealth and telemedicine services either only run on a specific operating system or only via a specific browser or end device.

*“Of course*,* it has to be available on Apple and Microsoft and all. Well*,* I can’t use an Android cell phone*,* for example. That you also offer these formats there and that you actually have a web interface*,* so all sorts of things and perhaps not just on the cell phone*,* but also on the computer.” (Patient #9)*.

Participants considered a lack of competence in using digital applications a barrier. If the use of eHealth and telemedicine is accompanied by a change of caregivers, i.e. therapists, this would lead to a lack of continuity of care.

*“That these can be different people*,* so to speak*,* that you then have several contacts and I don’t know whether I would tell someone I’m seeing for the first time now*,* when I’ve perhaps known my GP for 15 years. I might tell them something different or meet them differently than someone I’ve just seen for the first time via telemedicine.” (Patient #18)*.

The complete hindering factors are depicted in Table 4.

**Table 4:** Thematically structured categorical system for the former patients’s sample – hindering factors

Category	Subcategory	Quote
System level	• Insufficient network coverage	• *“A stable connection, otherwise it will be difficult.” (Patient #1)*
	• Software	• *“Can’t*,* so uploading the portal was possible from my side*,* but they couldn’t see it. Technical problem again.” (Patient #4)*
	• Hardware	• *“Because some practices are still very old-fashioned. With a fax machine*,* where I think to myself: Yes*,* even in the city*,* with my GP*,* they now have an e-mail address. What a miracle.” (Patient #15)*
	• Interface compatibility	• *“So of course*,* it has to be available on Apple and Microsoft and all sorts of things. I cannot operate an Android-based phone*,* for example.” (Patient #3)*
Patient level	• Poor digital literacy	• *“I’m trying to think of people who really have a problem with it and would make use of it*,* which would mainly be those who didn’t grow up in the digital age.” (Patient #19)*
	• Insufficient private information	• *“Then there’s also the whole IT security aspect*,* like where the app is managed or where it’s hosted. And things like that. Data protection.” (Patient #9)*
	• Lack of continuity of care	• *“And everything in our power is guaranteed here so that we can ensure and offer you good prevention and aftercare. If that is not the case*,* it will be hard to earn trust.” (Patient #3)*
	• Addressing negative findings	• *“So that you can call up findings or something like that. If it’s somehow positive*,* of course. Of course*,* if it’s somehow negative*,* then that’s not so good. Then you’re more likely to discuss it directly” (Patient #5)*
	• Low usability	• *“That could make it more difficult for me and if an app isn’t programmed well*,* that it crashes all the time*,* that’s of course also an obstacle*,* no*,* when does IT ever work in such a way that it works?” (Patient #10)*
	• Lack of face-to-face treatment	• *“That would be*,* for example*,* a video call with a doctor or simply transmitting the results face to face. That should not be missing.” (Patient #1)*
	• Language barriers	• *“The sentence should not contain three or four commas*,* so after two lines you should still know what you want. In the language I speak. (Patient #2)*

### Intervention identification

**Fig. 2:**
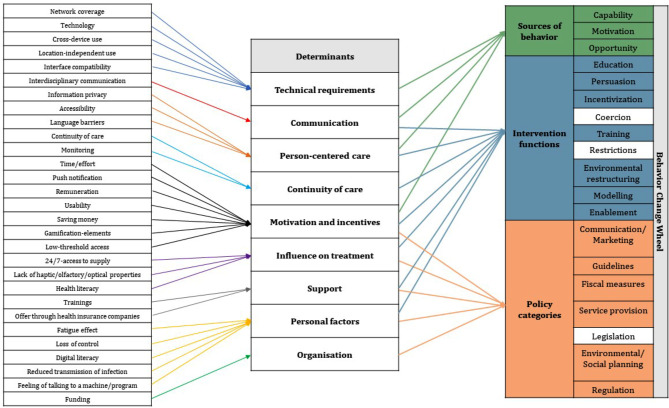
Assignment of the identified determinant categories to the aspects of the BCW [43]

Upon identification, themes were grouped into determinants, which were then mapped across the BCW model for designing behavior change interventions [43]. A comparison showed that all our identified factors could be found in the intervention categories of the established model, except for the categories *Coercion*, *Restrictions* and *Legislation*. The entire comparison can be seen in Fig. 2.

## Discussion

This study provides a timely view on the expectancies, beliefs and ideas of GPs and former cancer patients in respect to the introduction of telemedical care services for young cancer survivors. In this context, various specific possible eHealth functions were identified as well as distinct reservations regarding the use of eHealth and the types of eHealth to be potentially utilized. In addition, determinants were determined at the system level and on the part of the stakeholders involved, i.e. physicians and former patients.

The data shown here is a good addition to the information already available in this area, as previous research has highlighted gaps in eHealth and telemedicine, such as the sustainability of digital health services, the varying general satisfaction with eHealth services and the inability to reach certain age groups with digital means [53–57].

Other studies examined eHealth and telemedicine on specific factors and isolated target groups of oncologic patients, for instance regarding the acceptance of eHealth and telemedicine among older adults with cancer or the use of telehealth in gynecologic cancer care [58, 59]. However, these results aren’t generalizable and cannot be applied to the broader context of this domain due to their niche-specific approach. This applies in particular to studies on smartphone-based applications, whose publications either report on the development phase alone or tend to address a rather specific population, such as people suffering from colorectal cancer [60, 61]. Consequentially, not distinguishing between age cohorts or kinds of oncological disease, our results provide generalizable and essential data for the implementation of eHealth-based health care services. Only a few studies have used such a direct and application-oriented approach yet, recommending an investigation of the characteristics of potential users and corresponding smartphone applications [62].

A stronger shift in current research towards the evidence-based practice of implementation science may provide orientation towards a more efficient and effective integration of eHealth and telemedicine in this domain [63]. An identification of determinants and the development of implementation strategies represents the first step in such practice, which is not yet widespread [64]. However, the recent post-pandemic years gave a boost to digitization in general which has also benefited the adoption of eHealth-based care services and implementation-related research to bring the former into action [65, 66]. Still, this has to be taken with a grain of salt due to the aforementioned low long-term adoption of digital tools in healthcare [26].

Given the focus of implementation science on identifying facilitators and barriers, it is critical to explore stakeholders’ views and attitudes toward these determinants in the process of adopting eHealth for long-term cancer survivorship care [67]. As facilitators and barriers to eHealth itself have been identified in preceding studies, most of these factors also affect the adoption of eHealth for the medical follow-up of former cancer patients. As far as the facilitating factors are concerned, digital literacy of corresponding users, saving time, money and effort and a well-performing technological infrastructure were found to have a positive impact in current literature and were also cited in the interviews we conducted [68]. Recurrent themes for hindering factors are the lack of nonverbal communication, missing haptics and the absence of any olfactory perception, which has also been proposed by the participants in this study [69, 70]. Other factors, such as a resistance to change by healthcare providers or a presumed reluctance of patients were not brought up by our sample [71]. As for the literature on the determinants of eHealth for cancer care, there is a paucity of evidence and even less so on the facilitating and hindering factors of eHealth-based care services for the long-term follow-up of former cancer patients, let al.one young cancer patients. The literature for this specific domain comprises only a few publications, providing perspectives for future research endeavors or describing general satisfaction levels [72, 73]. To counteract the lack of evidence, our data and impact factors provide an application-oriented service towards the well-founded introduction of appropriate eHealth and telemedicine formats.

Lastly, as described in the existing literature, the transition phase plays an important role for this group of patients. This was also described in our interviews with general practitioners. Previous studies have shown that digital health formats have the potential to support patients in the transition phase, e.g. in the exchange of information between different practice teams and the patient [74]. In the future, it will be necessary to examine in detail how eHealth services can fill gaps in care.

eHealth and Telemedicine are about communication [75]. Accordingly, a major part of the factors brought up by the GPs and former patients we interviewed revolved around communicative themes.

While synchronous communication usually comes along with some kind of video consultation, live chat or phone call, asynchronous communication in this realm usually consists of exchanging mails or delayed text messages [76]. Current literature on the part of the direct care-related communication emphasizes the importance for patients of being able to quickly interact with their health care providers if needed, for instance with regard to risk assessments or psychosocial care [77]. As far as more general communication is concerned, existing publications in the domain of cancer treatment show that patients greatly appreciate receiving information about further treatment and guidance on organizational aspects from their physicians [78]. The main theme of our data revolved around the importance of ensuring that patients are connected to long-term care. Addressing this very topic, GPs of our sample mentioned the usefulness of web portals as well as smartphone applications for former patients to stay in touch. Especially the latter was considered impactful for the young target group of patients, since young people are associated with a broader and greater use of technologies [79]. Furthermore, they show a higher proneness to use digital means for information acquisition [80]. This is reinforced by the fact that young people use the Internet for many of their daily activities, including seeking help [81]. Thus, eHealth-based and telemedical care services may be an effective way of keeping young cancer survivors in long-term health care. Additionally, being given the very tools to self-organize and self-manage their own chronic health care by means of eHealth services may function as a huge support of the young patients’ self-efficacy and health literacy in the sense of an increased degree of *Patient Empowerment* [82–84]. This is also endorsed by comprehensive models for the health care of patients with chronic illnesses such as the Chronic Care Model, which aims to transform the daily care of patients suffering from chronic conditions from acute and reactive methods to proactive and planned ways of care services [85–87]. However, it is of importance to keep an eye on the potential drawbacks of eHealth and telemedicine, as genuine authenticity, a balance between professionalism and informality and the time between responses in digital communication formats have to be carefully balanced to express an appropriate degree of valuable factors such as empathy and active listening [88]. Also, for eHealth to be implemented, the corresponding infrastructure, such as an appropriate bandwidth for signal processing of telecommunication, has to be established [89]. This places especially less developed and rural regions at a disadvantage [90].

### Strengths and limitations

One strength of this study is the involvement of GPs as they play a crucial part in the follow-up care of young cancer survivors and are often the first contact for patients. Although GPs play an important role in guiding health care services for cancer patients, there are multiple medical disciplines involved in the overall care of cancer patients and survivors, that we did not take into account with our study [91]. Therefore, we cannot rule out the possibility that additional hypotheses may be identified if additional medical personnel are included in future research.

Furthermore, as for the qualitative content analysis, we could rely on a multidisciplinary research team consisting of a psychologist, a health services researcher, a speech and language therapist and two GPs. This constellation ensures the corresponding quality criterion of intersubjective comprehensibility of qualitative research and thus provides support for the validity of our data [92].

However, although diving into application-oriented depths, due to the methods used, this study stays at the level of generating hypotheses [93]. Therefore, our results should only be generalized, e.g. to other health care systems, with caution.

Lastly, while the gender ratio among former patients was balanced, the ratio among GPs was more unbalanced, with a slight underrepresentation of women compared to men.

## Conclusions

eHealth has the potential to provide patients as well as stakeholders with tools that increase their self-efficacy, access to care and ability to act.

In particular, issues of interconnectivity, data security, and business continuity need to be addressed.

Providing various forms of communication between patients and their caregivers and providing information that empowers self-management are critical to the successful implementation of eHealth and telemedicine applications.

## Electronic supplementary material

Below is the link to the electronic supplementary material.

Supplementary Material 1

Supplementary Material 2

Supplementary Material 3

## Data Availability

The datasets used during the current study are available from the corresponding author on reasonable request.
