# Supplementary material for: Determinants of the implementation of eHealth-based long-term follow-up care for young cancer survivors: a qualitative study
Source: BMC Cancer. 2024 Sep 18;24:1159. doi: 10.1186/s12885-024-12910-6 (PMC11411906; doi:10.1186/s12885-024-12910-6)
Supplement: Supplementary file 2 — Supplementary Material 2 [file 12885_2024_12910_MOESM2_ESM.pdf]

INTERVIEW GUIDE FOR QUALITATIVE INTERVIEWS ON LONG-TERM AFTERCARE

Interview content for semi-structured qualitative interviews with general practitioners, pediatricians and oncologists in private practice

- What is your experience in caring for children, adolescents and young adults with cancer?
- In your experience, what aspects would motivate this patient group to participate in such a study?
- What do you expect from a telehealth (e. g. app/videoconferencing) service for this patient group?
- In your experience, what factors inhibit the use of such telehealth services?
- In your opinion, what factors encourage the use of such telehealth services?
- In your experience, what are the barriers to guideline-based care?
- What factors do you think promote guideline-based care?
- What other aspect is important to you that we haven't talked about yet?

Contact person: Prof. Dr. med. Jost Steinhäuser
Institute of Family Medicine • Ratzeburger Allee 160 • 23538 Luebeck
