# Supplementary material for: Determinants of the implementation of eHealth-based long-term follow-up care for young cancer survivors: a qualitative study
Source: BMC Cancer. 2024 Sep 18;24:1159. doi: 10.1186/s12885-024-12910-6 (PMC11411906; doi:10.1186/s12885-024-12910-6)
Supplement: Supplementary file 3 — Supplementary Material 3 [file 12885_2024_12910_MOESM3_ESM.pdf]

UNIVERSITÄT ZU LÜBECK

UK SH UNIVERSITÄTSKLINIKUM Schleswig-Holstein

INTERVIEW GUIDE FOR QUALITATIVE INTERVIEWS ON LONG-TERM AFTERCARE

Interview content for semi-structured, qualitative interviews with children, adolescents, and young adults who have had cancer

- What experience have you had with the use of telehealth services (e. g. app/videoconferencing)?
- What expectations and wishes do you have for a telemedicine-supported care service (e. g. app/videoconferencing)?
- What factors would make it difficult for you to use a telemedicine-supported care service (e. g. app/videoconferencing)?
- What factors would make it easier for you to use a telehealth service?
- What are your reasons for participating in the study?
- What other aspect is important to you that we haven't talked about yet?

Contact person: Prof. Dr. med. Jost Steinhäuser
Institute of Family Medicine • Ratzeburger Allee 160 • 23538 Luebeck
